# Integrated Bioprocessing and Nanoencapsulation of Brewer’s Spent Grain Phenolic Compounds: A Sustainable Valorization Strategy

**DOI:** 10.3390/plants15192988

**Published:** 2026-09-30

**Authors:** Ingrid da Costa Maia, Carolina Thomaz dos Santos D’Almeida, Pedro Henrique Santos, Ana Bari Koifman, J. Vladimir Oliveira, Tamara Agner, Pedro Henrique Hermes de Araújo, Claudia Sayer, Juliana Furtado Dias, Elisa d’Avila Costa Cavalcanti, Mariana Simões Larraz Ferreira

**Affiliations:** 1 Food and Nutrition Graduate Program (PPGAN), Nutrition School, Federal University of the State of Rio de Janeiro (UNIRIO), Rio de Janeiro 22290-250, RJ, Brazil; ingridmaia@edu.unirio.br (I.d.C.M.); carolina.dalmeida@edu.unirio.br (C.T.d.S.D.); anabkoifman@edu.unirio.br (A.B.K.); juliana.dias@unirio.br (J.F.D.); 2 Center of Innovation in Mass Spectrometry, Laboratory of Protein Biochemistry (LBP-IMasS), Federal University of the State of Rio de Janeiro (UNIRIO), Rio de Janeiro 22290-250, RJ, Brazil; 3 Engineering, Modeling and Applied Social Sciences Center, Federal University of ABC, Santo André 09280-560, SP, Brazil; henrique.p@ufabc.edu.br; 4 Department of Chemical Engineering and Food Engineering (EQA), Federal University of Santa Catarina (UFSC), Florianópolis 88040-900, SC, Brazil; jose.vladimir@ufsc.br (J.V.O.); tamara.agner@ufsc.br (T.A.); pedro.h.araujo@ufsc.br (P.H.H.d.A.); claudia.sayer@ufsc.br (C.S.); 5 Laboratory of Microbial Biotechnology, Department of Biochemistry, Institute of Chemistry, Federal University of Rio de Janeiro (UFRJ), Rio de Janeiro 21941-909, RJ, Brazil; elisa@iq.ufrj.br

**Keywords:** *Aspergillus oryzae*, bioactive compounds, craft beer, nanostructured lipid carriers, solid-state fermentation, UHPLC–MS/MS

## Abstract

Brewer’s spent grain (BSG), the major by-product of the brewing industry, is an abundant lignocellulosic biomass rich in phenolic compounds, although their association with the cell wall matrix limits their recovery and functional application. This study aimed to enhance phenolic compound recovery from BSG by integrating solid-state fermentation (SSF) with *Aspergillus oryzae* and green extraction technologies and to stabilize the recovered compounds using nanostructured lipid carriers (NLCs). Fermentation was evaluated for up to 7 days. Fermented and unfermented BSG were subsequently subjected to pressurized liquid extraction (PLE) and microwave-assisted extraction (MAE) with ethanol at 50–150 °C. Phenolic recovery was assessed by reducing capacity, DPPH antioxidant activity, and targeted UHPLC–MS/MS profiling. A two-day SSF period was selected as optimal, increasing reducing capacity 5-fold and antioxidant activity 7-fold compared with unfermented BSG. PLE provided the most efficient extraction performance, particularly at 130–150 °C, while SSF further enhanced phenolic recovery, especially at intermediate extraction temperatures. Targeted analysis quantified 10 phenolic compounds and related compounds and revealed compound-specific responses to processing. Fermented BSG extracted by PLE at 150 °C showed the highest *p*-coumaric acid content (16.0 ± 0.07 µg/g BSG), whereas the maximum *trans*-ferulic acid content was reached after MAE at 130 °C (21.5 ± 0.09 µg/g BSG). Selected PLE extracts were successfully incorporated into carnauba wax-based NLCs, producing particles below 300 nm and encapsulation efficiencies of up to 35%. Bioprocessing integrated with green extraction substantially improved the recovery of bioactive compounds from BSG, while lipid-based encapsulation provided a promising strategy for their stabilization, supporting the sustainable valorization of this brewing by-product.

## 1. Introduction

Beer production has expanded significantly worldwide in recent years, and the Brazilian market has undergone notable consolidation, with 1847 legally registered breweries and a production exceeding 15 billion liters in 2024 [1]. However, this growth comes with the challenge of managing large quantities of brewing by-products, mainly brewer’s spent grain (BSG), which constitutes 85% of the total waste generated during beer production [2]. For every 100 L of beer brewed, around 20 kg of wet BSG are generated, most of which are currently used as animal feed or disposed of in landfills, strategies that are insufficient given the scale of global production [3]. As sustainability, renewable resource use, and the circular economy gain global importance, the valorization of this by-product for the agri-food, cosmetic, and pharmaceutical industries presents a strategic opportunity.

BSG consists primarily of the outer layers of barley grains, including the husk, pericarp, and seed coat, and is characterized by a high content of lignocellulosic fiber and proteins [4]. In addition to these components, BSG is also a valuable source of phenolic compounds (PCs), which contribute to its potential as a functional ingredient [3,5,6]. PCs are plant-specialized metabolites associated with antioxidant activity and multiple health-promoting effects, such as anti-inflammatory, antimutagenic, and anticancer properties [7]. However, in BSG, these compounds are predominantly bound to the plant cell wall matrix through covalent interactions, limiting their bioaccessibility [8]. Therefore, the application of bioprocessing strategies is essential to enhance the release and functional potential of these bioactive compounds.

Among these strategies, solid-state fermentation (SSF) stands out as a promising approach for BSG valorization due to its low water requirement, cost-effectiveness, and ability to promote the production and release of bioactive compounds. During SSF, filamentous fungi produce enzymes, e.g., β-xylosidase, β-galactosidase, α-amylases, cellulases, and feruloyl esterases [9,10]. These enzymes play a key role in disrupting the structural integrity of the lignocellulosic matrix, cleaving the bonds between PCs and cell wall components, thereby increasing their solubility and extractability [11]. Several studies have investigated the application of SSF on BSG compounds, demonstrating a mutually beneficial relationship between the microorganism and the substrate: BSG serves as an economical source of carbon and nitrogen for microbial growth, while filamentous fungi can enhance the phenolic content of BSG by up to sevenfold [6,11,12].

In addition to bioprocessing, the recovery efficiency of phenolic compounds is strongly influenced by the extraction technique used. Conventional solid–liquid extraction methods have been successfully applied to BSG [6]. Nevertheless, increasing attention has been directed toward alternative approaches based on green chemistry principles to reduce solvent consumption and improve extraction efficiency. In this regard, microwave-assisted extraction (MAE) and pressurized liquid extraction (PLE) have emerged as promising techniques for the sustainable recovery of bioactive compounds from BSG [13,14,15]. However, the use of high temperatures in these methods presents a dual effect: while thermally labile PCs may undergo degradation, bound phenolics can be released through thermal hydrolysis, enhancing their availability [16].

Once extracted, the practical application of phenolic-rich extracts remains limited due to their instability under environmental conditions—such as temperature and pH—and interactions with different matrices. In this context, nanoencapsulation has emerged as an effective strategy to improve the stability, solubility, and bioavailability of these compounds [17]. Carnauba wax, derived from *Copernicia prunifera* (the carnauba palm tree), is a particularly attractive wall material for encapsulating BSG compounds due to its biocompatibility, industrial acceptance, and ability to form stable lipid-based carriers [18]. In particular, nanostructured lipid carriers (NLCs) based on carnauba wax can provide protection against environmental degradation and enable controlled release of bioactive compounds, enhancing their functional performance [19].

Despite their potential, the effects of carnauba wax nanoparticles loaded with BSG compounds remain unexplored. This innovative approach seeks to promote the valorization of BSG, a by-product of the beer industry, while supporting sustainability initiatives. Such NLC systems may facilitate the incorporation of BSG-derived phenolic compounds into functional foods, nutraceuticals, and other bioactive formulations by improving their stability and enabling controlled delivery. In this context, this study aimed to achieve the following goals: (1) recover phenolic compounds from BSG using eco-friendly non-conventional extraction techniques, combined with SSF; (2) produce nanostructured lipid carriers (NLCs) containing these compounds through the melt dispersion technique, using carnauba wax as a carrier.

## 2. Results and Discussion

### 2.1. Selection of Fermentation Time in Phenolic Compounds Release

For preliminary analyses, fermentation kinetics were carried out to follow the antioxidant activity, based on the conventional extraction. SSF promoted a sharp release of phenolic compounds during the first two days of fermentation, resulting in a 5-fold increase in reducing capacity and a 7-fold increase in antioxidant activity compared to control unfermented BSG (Figure S2). This result is due to the catalytic enzymes produced by the filamentous fungus that break down the bonds between antioxidant compounds and the lignocellulosic matrix [6]. The autoclaved BSG, even before fermentation, also showed an interesting result: the intense temperature applied (i.e., 110 °C for 30 min) starts to promote the release of the antioxidant compounds. Beyond 48 h (days 3 to 7), these levels stabilized, indicating that 2 days of fermentation were sufficient to achieve phenolic release. These findings corroborate previous research, where SSF of agro-industrial waste increased antioxidant activity by 2.3 times, with the peak activity occurring within the first 2 days of fermentation [20]. Based on these results, a fermentation period of 2 days was selected for BSG in subsequent analyses.

### 2.2. Effect of the Extraction Temperature of Phenolic Compounds Using Unconventional Techniques

After establishing the optimal fermentation time, extraction conditions were subsequently optimized. The control unfermented BSG and 2-day fermented BSG samples were subjected to unconventional extraction methods (MAE and PLE) at different temperatures. Conventional extraction was used as a control. The obtained extracts were weighed to calculate the yield in each sample and were considered in the results (Figure S3). Antioxidant activity at each temperature, measured by the DPPH method, is presented in Figure S4 and expressed per g of extract (A) and per 100 g of BSG sample (B).

Overall, raising the extraction temperature had a positive effect on both fermented and unfermented BSG samples across both techniques, resulting in a progressive increase in extract yield (Figure S3) and antioxidant activity (Figure S4). At the lowest temperature (50 °C), unfermented BSG extracted by PLE exhibited nearly 10 times higher antioxidant activity, and by MAE, 11 times higher, compared to conventional extraction (Figure S4A), underscoring the greater efficiency of unconventional extractions.

As temperature increased, antioxidant activity in the extracts and extract yield continued to rise, enhancing the antioxidant activity relative to unfermented BSG. The high temperatures applied in both extraction techniques enhanced the action of the solvent, decreasing its viscosity and surface tension, increasing the solvent transport to the matrix sample, and accelerating the extraction of compounds [21].

In the 2-day fermented samples, SSF had a predominant effect on phenolic extraction, and the yield of the obtained extracts increased by an average of 25% for both extraction techniques (Figure S3). Compared to the control (unfermented), the yield of fermented BSG samples increased 1.5- and 2.0-fold with MAE-F and PLE-F, respectively (Figure S3). This result is expected, as SSF generates enzymes that facilitate biomass degradation, enhancing solvent penetration into BSG and thereby improving the accessibility and extractability of phenolic compounds [22]. While this progressive increase was evident in both methods, in MAE-F, the impact of fermentation on the extraction yield diminished at temperatures above 130 °C (Figure S3). This change is marked by an inflection point at 130 °C, from which the yield of MAE-F samples is lower than that of unfermented samples extracted by PLE.

The antioxidant potential of fermented samples exhibited a similar trend (Figure S4). SSF significantly enhanced the fermented BSG PLE antioxidant activity, showing higher activity at nearly all temperatures when compared to unfermented BSG PLE (Figure S4B). No significant difference (*p* > 0.05) was observed between fermented and unfermented BSG PLE at 150 °C (Figure S4B). For MAE, SSF similarly increased antioxidant activity; however, at extreme temperatures (50 °C and 150 °C; *p* > 0.05), SSF showed minimal impact on BSG. This indicates that SSF is an effective pretreatment for maximizing phenolic compound utilization in BSG, with the greatest efficiency observed at intermediate temperatures (70–130 °C) across both extraction methods. Based on the overall results of this preliminary temperature-screening step, 130 and 150 °C were selected as the most promising conditions for a more detailed comparison of MAE and PLE, as described in the following subsection.

### 2.3. Comparison Between Unconventional Extraction Methods of Phenolic Compounds

**Figure 1:**
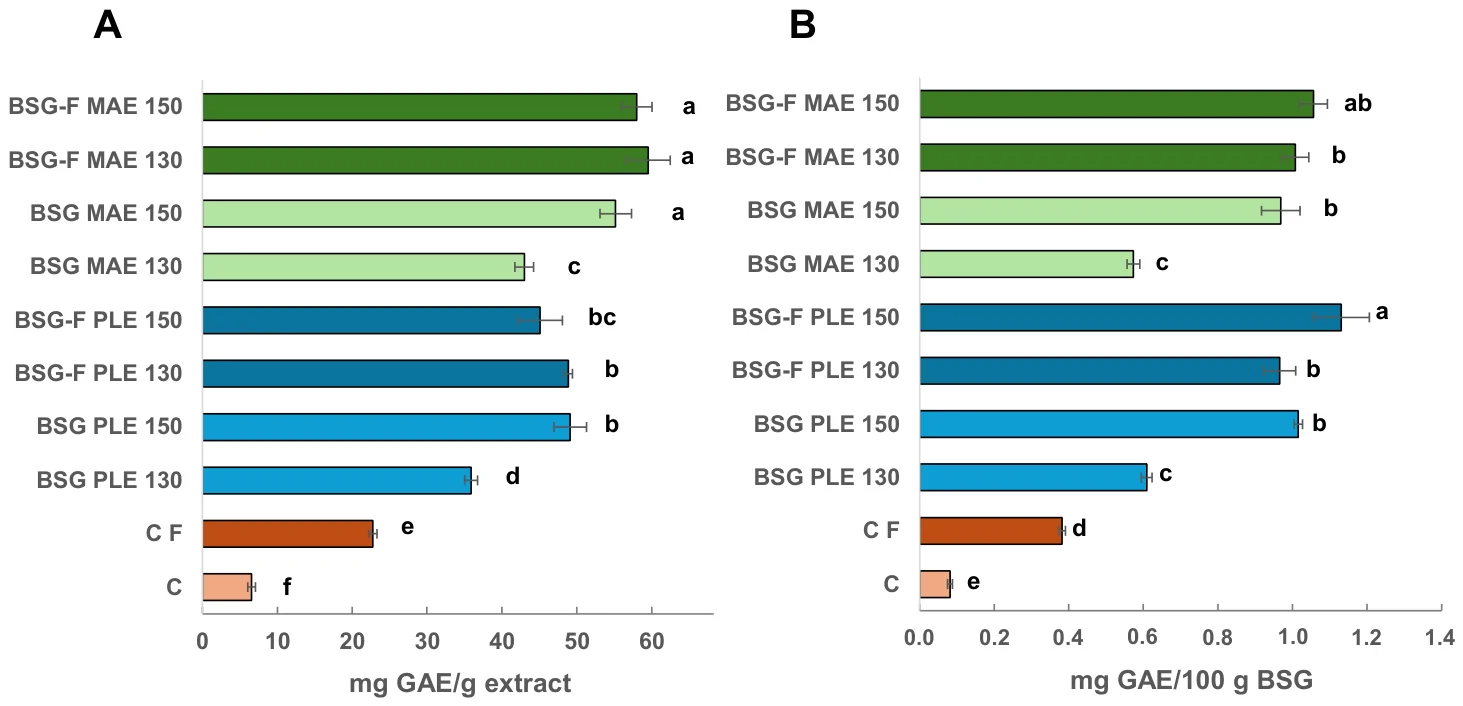
Reducing capacity of Folin–Ciocalteu is expressed as mg gallic acid equivalent (GAE) in relation to the extract (**A**) and in relation to BSG (**B**). Different letters indicate significant differences (*p* < 0.05). C: control; PLE: pressurized liquid extraction; MAE: microwave-assisted extraction; F: fermented. The values 130 and 150 correspond to the extraction temperature in °C.

Following the preliminary optimization steps described in Section 3.1 and Section 3.2, an SSF duration of 2 days and extraction temperatures of 130 and 150 °C were selected for further investigation. These selected conditions were subsequently evaluated to compare the performance of MAE and PLE in greater detail. TRC and DPPH antioxidant activity were considered both relative to the recovered extract and to the initial BSG mass, providing complementary information on the characteristics of the obtained extracts and the overall recovery from the original by-product. Under these selected conditions, PLE generally showed a slightly better performance than MAE, particularly in terms of extraction yield and antioxidant activity (Figure 1 and Figure S3).

Evaluating the total reducing capacity of the samples (Figure 1), there were no significant differences between MAE and PLE (*p* > 0.05), indicating that both extraction methods exhibited similar responses to temperature variations and the application of SSF pretreatment. Interestingly, this pattern differed from that observed for the antioxidant activity of the extracts (Figure S4), where PLE at 130 and 150 °C, before SSF (14% and 30%, respectively) and after SSF (37% and 53%, respectively), showed superior performance compared to MAE (Figure S4). These findings lead to two hypotheses: (1) the enhanced antioxidant capacity may be attributed to other compounds that were more effectively extracted via PLE; (2) PLE recovers a distinct phenolic profile than MAE, with these phenolic classes exhibiting greater antioxidant activity.

In addition, the literature highlights several operational benefits of PLE compared to MAE. PLE requires less solvent and shorter extraction times, making it more economical for industrial-scale applications. Moreover, PLE involves fewer operational steps, as most instruments are automated and produce a pre-filtered extract, which enhances reproducibility and reduces labor.

To sum up, PLE emerges as the most promising extraction method investigated in this study, supporting the hypothesis that it facilitates the extraction of phenolic compounds with enhanced antioxidant properties. To validate this hypothesis and to establish PLE as the selected extraction method for BSG, further analysis using more specific and sensitive techniques, such as UHPLC–MS/MS, is recommended.

### 2.4. Quantification of Phenolic Compounds by Targeted Metabolomics

**Figure 2:**
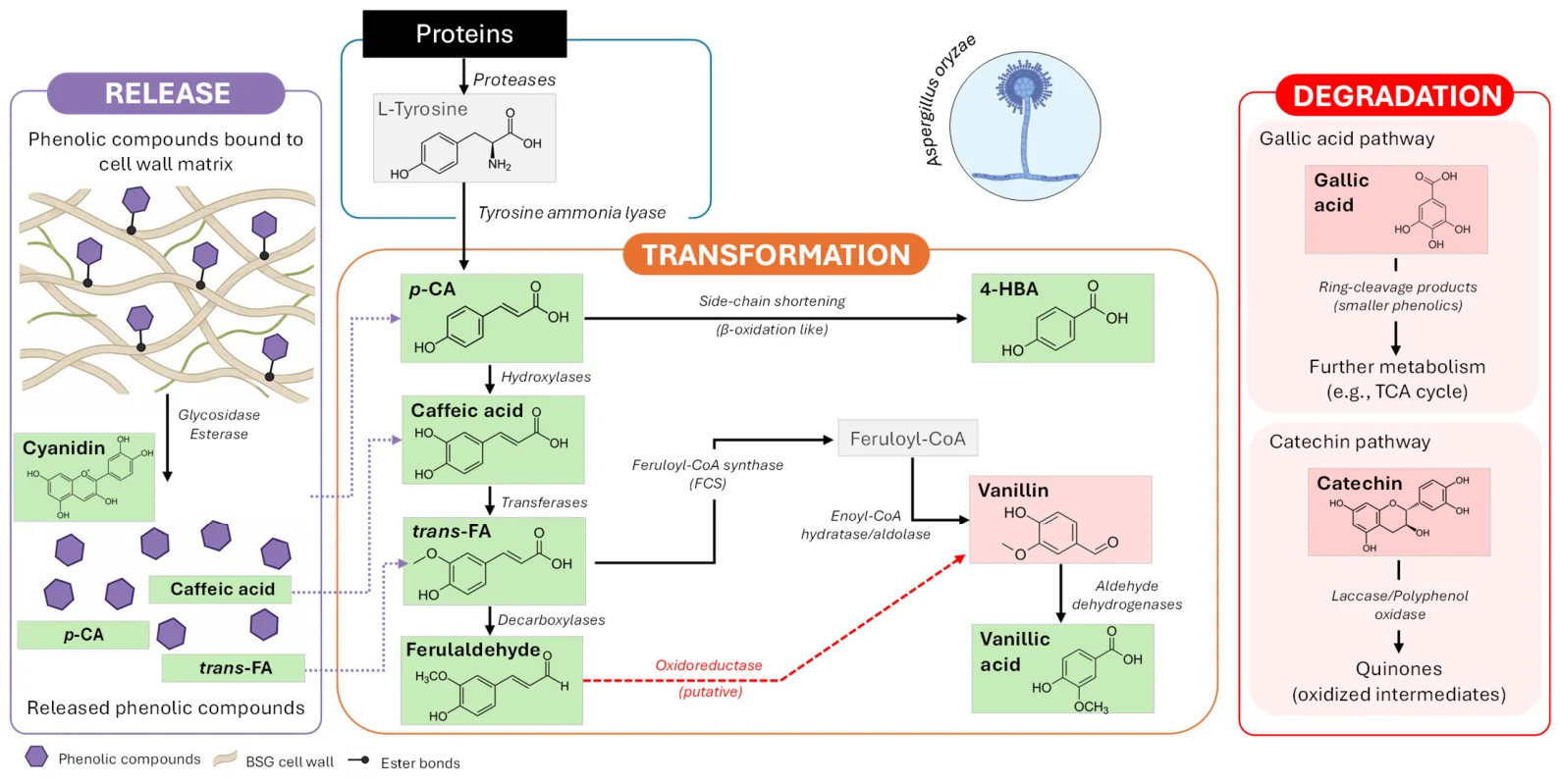
Proposed pathways for phenolic modulation in BSG during SSF, integrating enzymatic release, microbial transformation, and degradation processes. Compounds highlighted in green boxes increased after SSF, while those in red boxes decreased. Gray compounds represent intermediate metabolites (not detected) included to support the proposed pathways. These pathways were constructed based on the experimental trends observed in this study and on metabolic information from KEGG pathway databases (https://www.kegg.jp/). 4-HBA: 4-hydroxybenzoic acid; *p*-CA: *p*-coumaric acid; *trans*-FA: *trans*-ferulic acid.

Targeted UHPLC–MS/MS analysis enabled the detection and quantification of ten phenolic compounds across the different samples, encompassing hydroxycinnamic acids (*p*-coumaric, *trans*-ferulic, and caffeic acids), hydroxybenzoic acids (4-hydroxybenzoic, gallic, and vanillic acids), phenolic aldehydes (ferulaldehyde and vanillin), flavanols (catechin), and anthocyanidins (cyanidin) (Table 1). To facilitate the interpretation of the concentration changes observed after SSF, the quantified compounds were manually contextualized within established metabolic relationships available in the KEGG database. Based on the experimentally observed quantitative trends and established biochemical relationships, a schematic pathway was proposed to illustrate potential mechanisms underlying phenolic modulation during SSF (Figure 2). This representation should therefore be interpreted as a mechanistic framework rather than as direct evidence of metabolic pathway activity.

Among the quantified compounds, hydroxycinnamic acids showed a pronounced response to both SSF and assisted extraction (Table 1). In unfermented BSG, PLE and MAE substantially increased the recovery of *trans*-ferulic and *p*-coumaric acids compared with conventional extraction. For *trans*-ferulic acid, increases of 56- and 340-fold were observed for PLE and 49- and 96-fold for MAE at 130 and 150 °C, respectively. A similar pattern was found for *p*-coumaric acid, particularly at 150 °C, with increases of approximately 290- and 215-fold for PLE and MAE, respectively. Caffeic acid was not detected following conventional extraction but became quantifiable under assisted extraction conditions. These results demonstrate the strong influence of extraction conditions on the recovery of hydroxycinnamic acids from the BSG matrix.

SSF further modulated the concentrations of these compounds, although the magnitude of this effect depended on the subsequent extraction conditions. *Trans*-ferulic acid showed substantial increases after SSF, particularly under MAE at 130 °C (8.7-fold) and conventional extraction (69-fold), whereas a slight decrease was observed under PLE at 150 °C (−11.6%). A similar pattern was observed for *p*-coumaric acid, with more pronounced increases under milder extraction conditions (conventional extraction: 8.4-fold; PLE at 130 °C: +51.7%; MAE at 130 °C: +75.1%) and comparatively limited changes at 150 °C (PLE: +10.4%; MAE: +22.9%). Caffeic acid followed the same general trend, becoming detectable under conventional extraction only after SSF (0.10 ± 0.00 µg/g BSG) and increasing by up to threefold under PLE and MAE at 130 °C, while remaining relatively stable under the more severe extraction conditions.

Mechanistically, these changes are primarily consistent with the enzymatic deconstruction of the lignocellulosic matrix during SSF (Figure 2). In BSG, hydroxycinnamic acids, particularly ferulic and *p*-coumaric acids, occur predominantly esterified to arabinoxylans and other structural polysaccharides [23]. During SSF, *A. oryzae* produces a broad enzymatic system, including esterases and carbohydrate-active enzymes such as xylanases, capable of disrupting the polysaccharide network and hydrolyzing linkages between phenolic acids and cell-wall components [24]. This process increases the accessibility of matrix-associated phenolics and consequently facilitates their subsequent recovery.

Interestingly, the relative effect of SSF was generally more pronounced following conventional extraction and assisted extraction at 130 °C than under the more severe conditions at 150 °C. This behavior suggests an interaction between biological and physicochemical matrix disruption. Under milder extraction conditions, the structural modifications promoted by fungal growth may play a greater role in determining phenolic accessibility. Conversely, at higher extraction temperatures, thermal and physicochemical disruption of the BSG matrix may itself promote substantial phenolic release, thereby reducing the relative contribution of the preceding SSF treatment.

Hydroxybenzoic acids showed a comparable response. In unfermented BSG, PLE and MAE were substantially more effective than conventional extraction, with concentrations reaching up to 78-fold higher for 4-hydroxybenzoic acid and 175-fold higher for vanillic acid under PLE at 150 °C (Table 1). Following SSF, both compounds generally increased across the evaluated conditions. As observed for hydroxycinnamic acids, the relative effect of SSF was less pronounced under more severe extraction conditions; under PLE at 150 °C, increases were limited to 11.1% for 4-hydroxybenzoic acid and 15.8% for vanillic acid.

In addition to enzymatic release, microbial transformation of phenolic compounds may contribute to the compositional changes observed after SSF (Figure 2). Once released from the matrix, hydroxycinnamic acids may undergo side-chain modification and shortening reactions, potentially contributing to the formation of structurally related hydroxybenzoic acids and phenolic aldehydes [25]. Ferulic acid, for example, can participate in microbial transformation routes involving intermediates such as ferulaldehyde and vanillin, with subsequent oxidation yielding vanillic acid through reactions involving oxidoreductases and aldehyde dehydrogenases [26]. Although the present study did not directly assess enzymatic activity or metabolic flux, the simultaneous changes observed among these structurally related compounds are consistent with the occurrence of phenolic biotransformation during SSF. Accordingly, the relationships depicted in Figure 2 represent plausible transformations supported by the quantitative trends and established biochemical reactions rather than experimentally confirmed conversion pathways in the present system.

In contrast, catechin, vanillin, and gallic acid generally decreased after SSF (Table 1), demonstrating that fungal treatment did not uniformly increase the concentration of all phenolic compounds. Catechin showed a consistent reduction across most extraction conditions, suggesting greater susceptibility to transformation or degradation during SSF. Vanillin and gallic acid displayed a similar overall tendency, although condition-specific increases were observed, particularly following conventional extraction and PLE at 150 °C. Ferulaldehyde exhibited a distinct behavior, being detected only following conventional extraction of fermented BSG. Its restricted detection may be consistent with its occurrence as a transient intermediate in phenolic transformation pathways [27], although its measured concentration may also be influenced by extraction conditions and chemical stability.

Cyanidin also showed a distinct response to SSF and extraction. While it was not detected in unfermented BSG following PLE or MAE, it became detectable after SSF under both extraction techniques, reaching higher concentrations at 150 °C. This behavior suggests that fungal modification of the BSG matrix increased the accessibility of anthocyanin-derived compounds or related structures. However, considering the susceptibility of anthocyanidins to processing conditions and structural transformations, these changes should be interpreted primarily as alterations in extractability and chemical recovery rather than direct evidence of de novo formation during SSF.

The decrease observed for some phenolics may result from microbial metabolism and oxidative reactions occurring during SSF. Once released from the matrix, phenolic compounds become more accessible not only for subsequent extraction but also for enzymatic transformation. Oxidative enzymes produced by filamentous fungi, including laccases and polyphenol oxidases, may promote oxidation of phenolic substrates, generating reactive quinone intermediates that can undergo polymerization, condensation, ring-cleavage, or further degradation [27]. Therefore, the final phenolic profile of fermented BSG likely reflects a dynamic balance between enzymatic release from structural components and subsequent transformation or degradation of the liberated compounds.

**Figure 3:**
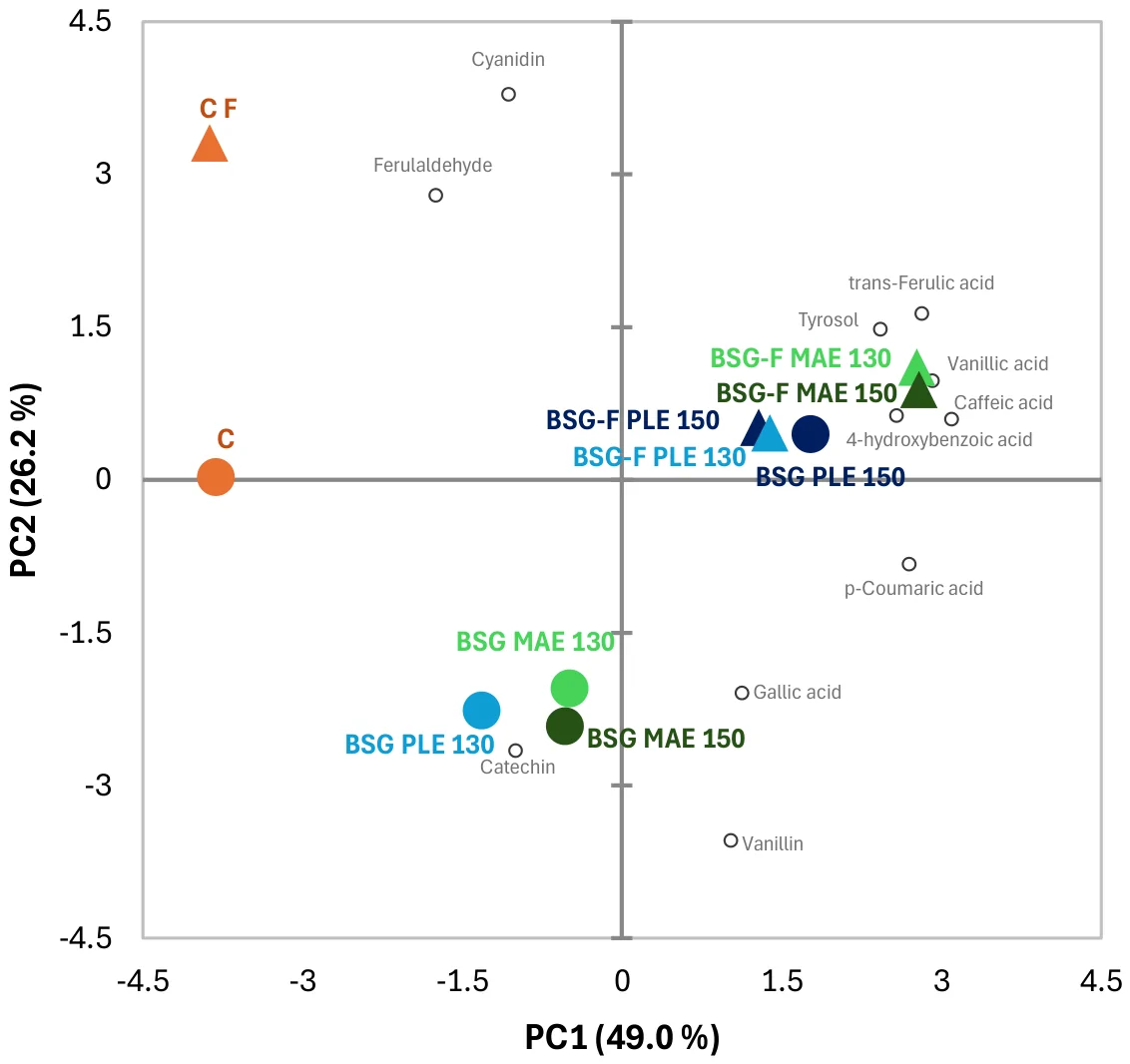
Principal component analysis (PCA) biplot of phenolic compounds in brewer’s spent grain (BSG) samples under different extraction conditions (C: control; PLE: pressurized liquid extraction; MAE: microwave-assisted extraction) and fermentation status (BSG: unfermented BSG; BSG-F: fermented BSG). Samples (symbols) are distributed according to the concentrations of identified phenolic compounds (variables).

These combined effects of SSF and extraction were further supported by multivariate analysis (Figure 3). PCA revealed a clear separation among samples (PC1 + PC2 = 71.2%), demonstrating that both biological treatment and extraction conditions contributed substantially to sample differentiation. Fermented and unfermented samples were clearly distinguished, particularly within MAE and PLE conditions, confirming SSF as an important driver of phenolic variability. Fermented samples were associated with higher concentrations of *trans*-ferulic, *p*-coumaric, caffeic, vanillic, and 4-hydroxybenzoic acids, whereas unfermented BSG samples were more strongly associated with catechin, vanillin, and gallic acid. The conventional extracts showed a distinct pattern, with fermented BSG positioned closer to cyanidin and ferulaldehyde. Collectively, these results indicate that SSF substantially modifies the accessibility and distribution of phenolic compounds even when followed by mild extraction.

Overall, the targeted metabolomic results demonstrate that SSF and extraction strategy jointly shape the phenolic profile recovered from BSG. SSF acts as a biotechnological pretreatment capable of modifying the lignocellulosic matrix, increasing the accessibility of matrix-associated phenolics, and simultaneously promoting transformation or degradation of released compounds. The subsequent extraction step determines the extent to which these modifications are reflected in the recovered phenolic fraction. The greater relative effect of SSF under milder extraction conditions further suggests that biological and physicochemical treatments may partially converge in their ability to disrupt the BSG matrix. Among the evaluated approaches, PLE showed a more consistent performance across extraction conditions, preserving the phenolic profile (Section 3.4) and antioxidant capacity (Section 3.3). This stability supports its selection for subsequent encapsulation, as it provides a more reliable recovery of phenolic compounds.

### 2.5. Nanostructured Lipid Carriers

Preliminary tests were performed without the extracts to determine the formulation and ultrasonic emulsification conditions for the nanoencapsulation experiment (Table S2). Six miniemulsion formulations (Mini) were tested. The first tested formulation (Mini-1) used an emulsion consisting of 1 g of carnauba wax (without Crodamol and without final cooling). This formulation exhibited a high polydispersity index (PDI) (Table S2), suggesting that the solidification process might have been incomplete, leading to particle aggregation and coalescence. Consequently, a new nanoparticle solidification method was implemented, in which the miniemulsion was immediately poured into 90 mL of deionized water at 4 °C after emulsification. Additionally, the liquid lipid Crodamol was incorporated to lower the melting point of the lipid mixture, helping to prevent premature solidification [28]. The revised solidification method and modified organic phase composition (Mini-2) decreased the PDI, though it remained above the target threshold (≥0.2). Increasing sonication time to 120 s (Mini-3) achieved the desired values, with further increases, up to 210 s (Mini-4 and Mini-5), leading to no additional improvements. However, raising the amplitude to 70% (Mini-6) significantly reduced both particle size and PDI compared to Mini-3, establishing these conditions as optimal for encapsulation tests. Based on these preliminary findings, the Mini-6 formulation was selected to produce NLCs with the PLE extracts: unfermented BSG (150 °C) and fermented BSG (130 °C).

After selecting the emulsion composition, BSG extracts were incorporated into the NLCs through simple and double emulsion (Table 2). The extract-containing NLCs showed slightly larger particle sizes when prepared by melt dispersion with double emulsification than by simple emulsification. In all cases, particle sizes remained below 300 nm with narrow size distributions. To enhance encapsulation of the extract’s phenolic compounds—which was around 30% in the simple emulsion—several new strategies were tested. For instance, adding soy lecithin to the lipidic phase (SE+L), which acts as a stabilizer by reducing the migration of hydrophilic compounds from the lipid to the aqueous phase, did not significantly impact encapsulation efficiency. Similarly, reducing the extract concentration in the simple emulsion (SE-E) showed no significant effect.

The double emulsion technique aimed to create a water reservoir within the lipid nanoparticles, enabling hydrophilic compounds to diffuse and remain inside the carriers. However, the encapsulation efficiency with this technique was similar to that of simple emulsion. Despite the lower encapsulation efficiency compared to other extract nanoencapsulations [29], the bioactive properties of the non-encapsulated extract—free in the aqueous phase—remain accessible as antioxidants and retain their health-promoting properties. Therefore, when NLCs are used as a colloidal dispersion, including the aqueous phase in which they were obtained, these properties function alongside the encapsulated extract, which enables controlled release in the gastrointestinal tract. It is important to note that encapsulation efficiency was assessed based on phenolic compound quantification, and the presence of other compounds in the extract may have different solubilities (phase partition coefficients), which could affect encapsulation efficiency depending on the basis used for calculation.

**Figure 4:**
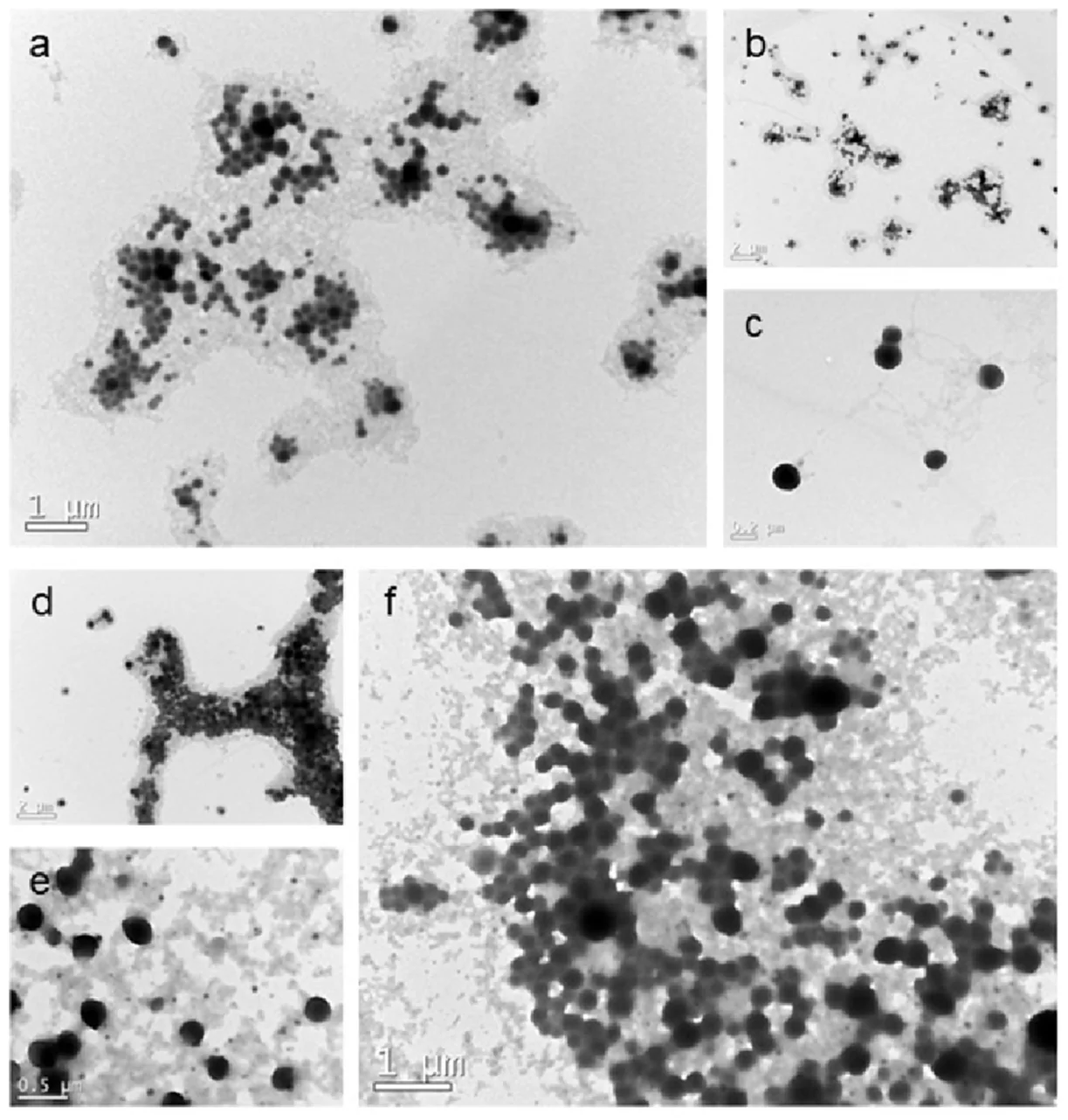
TEM micrographs of nanostructured lipid carriers containing unfermented brewer’s spent grain extracts, prepared using (**a**–**c**) melt dispersion technique and (**d**–**f**) the double emulsion/melt dispersion technique.

The nanometric size range of NLCs, with diameters consistent with those measured by DLS, was confirmed by TEM images (Figure 4). Simple and double emulsion samples with unfermented BSG extracts were tested. The NLC exhibited a narrow size distribution, spherical shape, smooth surface, and uniform appearance, indicating that no core–shell morphology was formed, with Crodamol and extract uniformly distributed within the lipid matrix. This morphology is typical for NLC formulations composed of a mixture of solid and liquid lipids [28].

**Figure 5:**
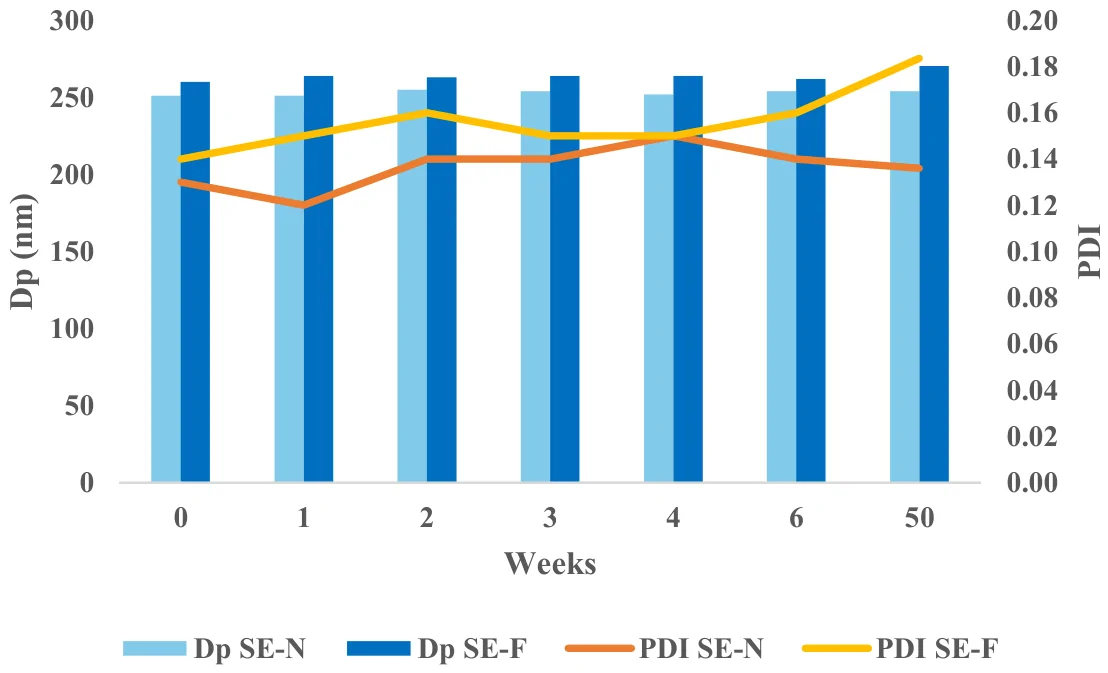
Values of average particle diameter (Dp) and polydispersity index (PDI) over time for NLCs obtained by simple emulsion (SE). Values represent the average of duplicate experiments. N—unfermented extract; F—fermented extract.

Finally, Dp and PDI were measured over time, and all NLCs prepared by SE demonstrated good stability (Figure 5), as did those prepared by DE. Particle size remained consistent with no significant changes in dispersion, indicating no destabilization for at least 50 weeks. Stability is an important factor in determining the shelf life of a product containing the emulsion [29]. A longer shelf life enhances the product quality and safety, emulsion efficacy, and consumer appeal, maintaining the product’s integrity and performance over time.

## 3. Materials and Methods

### 3.1. Materials

Pilsen-type BSG sample was kindly provided by a craft brewery (Brewpoint Brewery, Petrópolis, RJ, Brazil). Carnauba wax T4 (CW) was kindly donated by GM Ceras (São Paulo, Brazil). Crodamol GTCC (medium-chain triglycerides from coconut oil) and soy lecithin (90%) were purchased from Alpha Química and Alfa Aesar (Haverhill, MA, USA), respectively. Polysorbate 80 (Tween 80) and analytical standards were obtained from Sigma-Aldrich (St. Louis, MO, USA). Milli-Q water was used in all experiments.

### 3.2. Brewer’s Spent Grain Preparation and Solid-State Fermentation

BSG samples were previously dried in a forced-air ventilation oven (MA 035, Marconi, Brazil) at 65 °C for 8 h and adjusted to approximately 70% moisture content. SSF was performed using *Aspergillus oryzae* (NRRL 32657), obtained from the Microbial Biotechnology Laboratory collection (UFRJ, Rio de Janeiro, Brazil). The autoclaved BSG was inoculated with 10^7^ spores/g and incubated at 30 °C [6]. Fermentation kinetics were monitored at predetermined intervals (0, 1, 2, 4, and 7 days). At each time point, samples were collected, dried (65 °C, 6 h), and stored at 4 °C until analysis. Autoclaved BSG was used as the control for basal PC concentration. Total reducing capacity (TRC) and antioxidant activity (DPPH assay) were determined in triplicate (Section 2.4) to define the optimal fermentation time. Hereafter, the samples are referred to in the text as unfermented BSG and fermented BSG to facilitate interpretation. For conciseness in figures and tables, BSG and BSG-F denote unfermented and fermented brewer’s spent grain, respectively.

### 3.3. Phenolic Compounds Extraction

PCs were extracted from unfermented BSG and 2-day fermented BSG, as selected in Section 3.1. The experimental design aimed to evaluate the effects of SSF and extraction techniques at different temperatures on the recovery of antioxidant compounds from BSG. Two extraction techniques were investigated independently: microwave-assisted extraction (MAE) and pressurized liquid extraction (PLE). Each technique was evaluated at six temperatures (50, 70, 90, 110, 130, and 150 °C), using 100% ethanol as the solvent. Conventional extraction at 25 °C was included as a mild reference condition rather than as a third method for direct temperature-matched comparison. The 50–150 °C range was specifically investigated for MAE and PLE because temperature is a critical operational parameter of these technologies, allowing the evaluation of the balance between enhanced extraction efficiency and the potential degradation of thermolabile phenolic compounds under more severe conditions. For all extraction procedures, a constant sample-to-solvent ratio of 1:20 (*w*/*v*) was maintained.

Non-conventional extractions—MAE and PLE extractions were performed in duplicate according to [30], with minor modifications. For MAE, 1 g of sample was extracted with ethanol using a microwave reactor (Monowave TM 200, Anton Paar, Graz, Austria) for 20 min at the selected temperature. For PLE extraction, 5 g of sample was placed in a 90 mL extraction vessel, with the remaining volume filled with layers of cotton and glass beads. Ethanol was delivered using an HPLC pump (Waters, Model 515, Milford, MA, USA) through a preheating vessel and subsequently through the extraction vessel. After reaching 100 bar, extraction was conducted in continuous mode at a flow rate of 5 mL/min using 100 mL of ethanol.

Conventional extraction—conventional extraction was performed as a control, according to [31] with modifications. Briefly, 200 mg of sample was manually macerated with 10 mg of Celite and extracted with 4 mL of ethanol (100%) at 25 °C for 20 min under agitation (Multi Bio RS-24, Biosan, Brazil). Samples were centrifuged (5000× *g*, 10 min, 25 °C), and the extraction was repeated twice; the supernatants were combined.

Extraction yield and antioxidant activity were used to evaluate the influence of temperature and extraction technique. Antioxidant activity was determined in triplicate by using the DPPH assay described in Section 2.4. Based on these results, the most promising extraction temperatures were selected for subsequent comparison of extraction techniques and targeted UHPLC–MS/MS phenolic quantification.

All extracts were evaporated (Fisatom, Model 801, SP, Brazil), weighed, and stored in amber tubes at −18 °C. Extracts were resuspended in ethanol (final concentration of 5 mg/mL for samples), filtered through a 0.22 µm nylon membrane, and stored under the same conditions.

### 3.4. Total Reducing Capacity and Antioxidant Activity

TRC was determined using the Folin–Ciocalteu method adapted to a microplate format according to [32]. Briefly, 100 µL of appropriately diluted extract was mixed with 50 µL of Folin–Ciocalteu reagent, followed by 150 µL of sodium carbonate solution (20%). After incubation for 30 min at 40 °C in the dark, absorbance was measured at 750 nm using a microplate reader (FlexStation III, Molecular Devices, CA, USA). Gallic acid was used for calibration (0–260 mg/L; R^2^ > 0.999), and results were expressed both as milligrams of gallic acid equivalents per gram of extract (mg GAE/g extract) and per 100 g of BSG on a dry basis (mg GAE/100 g BSG).

Antioxidant activity was evaluated using the DPPH radical-scavenging assay according to [33], with adaptation to a microplate reader assay. Briefly, 20 µL of appropriately diluted extract was mixed with 280 µL of DPPH solution at 80 µM, prepared in methanol. The reaction mixture was incubated for 30 min at 25 °C in the dark, after which absorbance was measured at 517 nm (FlexStation III, Molecular Devices). Trolox was used as the calibration standard (0–2 µg/mL, R^2^ > 0.999). Results were expressed in µmol of Trolox equivalent per gram of extract (µmol TE/g extract) and per 100 g of BSG on a dry basis (µmol TE/100 g BSG).

### 3.5. Targeted UHPLC–MS/MS Analysis

The quantification of PCs in BSG extracts was performed using an ultra-performance liquid chromatography system coupled to a triple quadrupole mass spectrometer (UHPLC–MS/MS) (ACQUITY UPLC I-Class and Xevo TQ-S, Waters Corp., Milford, MA, USA). Chromatographic separation was performed on an HSS T3 column (2.1 × 150 mm, 1.8 μm) at 40 °C, with the autosampler maintained at 8 °C. The mobile phases consisted of water (A) and acetonitrile (B), both containing 0.3% (*v*/*v*) formic acid, and were subjected to the following processes: 0–10.00 min, 5–27.5% B; 10.00–12.50 min, 27.5–55% B; 12.50–13.50 min, 55–100% B; 13.50–16.50 min, 100% B; re-equilibration to 5% B until the 20.00 min mark. The flow rate was 0.3 mL/min, and the injection volume was 5 μL.

Mass spectrometric detection was performed using electrospray ionization (ESI) in both negative and positive modes, operating in multiple reaction monitoring (MRM). Instrument parameters were optimized using IntelliStart™ and further refined manually. The desolvation gas temperature and flow were set at 500 °C and 1000 L/h, respectively, and the capillary voltage was 2.0 kV.

MRM transitions were initially selected based on established fragmentation information available in spectral databases (MassBank and MoNA) and, when available, on parameters provided by the Waters Quanpedia database. The transitions, cone voltages, and collision energies were subsequently evaluated and optimized using authentic analytical standards on the Xevo TQ-S system. Final quantifier and qualifier transitions were selected based on signal intensity and selectivity, including discrimination among structurally related compounds when applicable. Retention times, optimized transitions, and calibration ranges are provided in Table S1. External calibration curves showed good linearity for all analytes (R^2^ > 0.999). Data acquisition and processing were performed using TargetLynx™ XS (version 4.2, Waters), with peak integration based on extracted ion chromatograms. Automatic integration was followed by manual inspection to ensure consistency. Quantification was carried out using the external standard method, and results were expressed as mean ± standard deviation of duplicate analyses.

Based on the concentration changes observed for the compounds quantified by targeted UHPLC–MS/MS, a schematic metabolic pathway was proposed to contextualize the effects of SSF on phenolic metabolism. The experimentally quantified compounds were manually positioned within an established metabolic pathway available in the Kyoto Encyclopedia of Genes and Genomes (KEGG) database. Known intermediate metabolites were included when necessary to represent established metabolic connections, even when they were not detected in the present study.

### 3.6. Nanoencapsulation with Lipid Carriers by Melt Dispersion Technique

Simple emulsion

NLCs containing the BSG extracts were prepared by the melt dispersion technique [28], with some modifications. The surfactant Tween 80 (3% in relation to the water, *w*/*w*) was dissolved in deionized water (10 g). The lipid phase (1 g) was prepared by combining carnauba wax (CW) and Crodamol under six different conditions varying time and ultrasonic amplitude (Table S2). To increase the extract encapsulation in the simple emulsion (SE), the addition of 0.01 g of soy lecithin (1% in relation to the lipid phase, *w*/*w*) in the lipidic phase was evaluated (SE+L). The aqueous and lipid phases were magnetically stirred at 95 °C, ~10 °C above the melting point of the CW (82.40 °C) (Figure S1, Table S3), until complete melting. The alcoholic BSG extract solution (5 mL, 10 mg/mL) was previously heated to 75 °C to avoid quick ethanol evaporation and then mixed with the lipid phase. Both phases were pre-emulsified with vigorous magnetic stirring for 3 min. The coarse emulsion formed was sonicated under heating conditions for 1 min (30 s on/10 s off) at 70% amplitude (VC 505 Vibra-Cell Ultrasonic Processor, Sonics & Materials; tip probe size of ½ in; Newtown, CT, USA). Finally, the resulting miniemulsion was poured into cold water (90 mL at 4 °C) to promote the rapid solidification of wax nanoparticles. An emulsion with half the concentration of extract (5 mL, 5 mg/mL) was also evaluated (SE-E).

Double emulsion

Double emulsion (DE) was prepared using a lipid phase (1 g) containing CW or a mixture of CW and Crodamol, melted under the same conditions as SE. Soy lecithin was added (1% in relation to the lipid phase, *w*/*w*) as a stabilizer. The internal aqueous phase (0.5 mL of deionized water, W1) was heated at the same temperature. The BSG extract (5 mL, 10 mg/mL) was heated to 75 °C and then mixed with the lipid phase at 95 °C. The W1 aqueous phase was added under magnetic stirring (15 min) to form a coarse emulsion that was sonicated for 30 s at 70% amplitude. Immediately after obtaining the W1/O emulsion, an external aqueous phase (10 g of deionized water and 3% Tween 80 in water, W2), preheated to the same temperature, was added and ultrasonically emulsified (30 s on/10 s off) at 70% amplitude to obtain the DE (W1/O/W2).

Then, the double miniemulsion was poured over 90 mL of cold deionized water (4 °C), which allowed the nanoparticles to solidify, avoiding possible aggregation or coalescence during the process.

### 3.7. Characterization of Nanostructured Lipid Carriers

Intensity-weighted average diameters of the NLC (Dp) and the polydispersity indices (PDIs) were measured by Dynamic Light Scattering (DLS; Zetasizer Nano S, Malvern Instruments, Malvern, UK) at 25 °C using a glass cuvette. Right after preparation, the colloidal dispersions were diluted further 1:10 (*v*/*v*) with deionized water before analysis. Results are expressed as the average of two experiments. The stability of the nanoparticles over time was assessed by size measurements, for a period of up to 50 weeks. The long-term colloidal stability of the dispersion, stored at 4 °C, was evaluated through nanoparticle size measurements over a 50-week period.

The morphology of the NLC was examined using a Transmission Electron Microscopy (TEM) instrument operating at 80 kV (JEM-101, Tokyo, Japan). Each sample was diluted to 0.1% in deionized water (*w*/*w*), and a droplet was then deposited onto a carbon-coated 400-mesh copper grid and allowed to dry at ~25 °C.

### 3.8. Encapsulation Efficiency

The BSG extract encapsulation efficiency (EE) was determined by measuring TRC by Folin–Ciocalteu [32] (please refer to Section 2.3) of the unencapsulated extract in the aqueous phase of the NLC dispersion. For this analysis, right after preparation, the NLCs were separated from the aqueous phase by centrifugation using an Amicon^®^ Ultra filter (0.5 mL, 100 kDa, Merck) at 13,400 rpm for 30 min (Centrifuge MiniSpin, Eppendorf). Then, an aliquot of the filtrate was analyzed, and the encapsulation efficiency (EE) was quantified by Equation (1):

(1) $$\mathrm{EE}\;\left(\%\right)=\left(1-\frac{\mathrm{TRC}2}{\mathrm{TRC}1}\right)\times 100$$

where TRC2 is the TRC found in the aqueous phase after centrifugation, and TRC1 is the initial TRC in the extract at the same dilution as the nanocarrier system [34].

### 3.9. Statistical Analysis

Statistical analyses were performed using XLSTAT 2025.2.0 software. Results are expressed as mean ± standard deviation of technical replicates. Differences among groups were assessed by one-way analysis of variance (ANOVA), followed by Tukey’s post hoc test, adopting a significance level of 5% (*p* < 0.05). In addition, multivariate analyses were conducted to further explore patterns and relationships within the dataset.

## 4. Concluding Remarks

This study demonstrates that BSG valorization can be enhanced by integrating complementary sustainable technologies, including bioprocessing, green extraction, and lipid-based delivery systems. Solid-state fermentation with *Aspergillus oryzae* improved the bioaccessibility of phenolic compounds in the BSG matrix, while MAE and PLE provided efficient and complementary approaches for their recovery. Targeted phenolic quantification showed that fermentation and unconventional extraction techniques not only increased the recovery of reducing compounds but also selectively reshaped their phenolic composition.

The subsequent incorporation of selected extracts into carnauba wax-based nanostructured lipid carriers demonstrated the feasibility of coupling phenolic recovery with stabilization for potential applications in emulsified foods, edible coatings, active packaging, and formulations designed to extend the shelf life of plant- and animal-based products. Although the encapsulation efficiency remained moderate and further formulation refinement is required, the obtained systems showed suitable nanoscale characteristics and long-term physical stability. Taken together, these findings support an integrated biorefinery-oriented approach in which fermentation, sustainable extraction, and nanoencapsulation can be combined to convert BSG from an abundant brewing by-product into a potential source of value-added bioactive ingredients. Future studies should focus on improving phenolic retention within the carriers and evaluating their release, bioaccessibility, and performance in food and nutraceutical matrices.

## Figures and Tables

**Table 1:** Quantification of phenolic compounds in brewer’s spent grain (BSG) extracts obtained under different extraction conditions.

Sample	T (°C)	4-HBA (µg/g BSG)	Caffeic Acid (µg/g BSG)	Catechin (µg/g BSG)	Cyanidin (µg/g BSG)	Ferulaldehyde (µg/g BSG)	Gallic Acid (µg/g BSG)	*p*-CA (µg/g BSG)	*trans*-FA (µg/g BSG)	Vanillic Acid (µg/g BSG)	Vanillin (µg/g BSG)
C	-	0.03 ± 0.00 ^h^	nd	0.19 ± 0.02 ^ab^	3.41 ± 0.06 ^e^	nd	0.01 ± 0.00 ^f^	0.05 ± 0.01 ^g^	0.05 ± 0.00 ^i^	0.03 ± 0.01 ^f^	0.05 ± 0.01 ^e^
C-F	-	0.18 ± 0.00 ^g^	0.10 ± 0.00 ^d^	0.02 ± 0.00 ^d^	9.09 ± 0.17 ^a^	0.02 ± 0.00 ^a^	0.05 ± 0.00 ^f^	0.42 ± 0.00 ^g^	3.46 ± 0.03 ^g^	0.33 ± 0.01 ^ef^	0.08 ± 0.01 ^e^
BSG PLE	130	0.83 ± 0.02 ^e^	0.14 ± 0.00 ^d^	0.22 ± 0.02 ^a^	nd	nd	0.72 ± 0.02 ^de^	4.89 ± 0.05 ^f^	2.80 ± 0.02 ^h^	1.25 ± 0.00 ^e^	3.68 ± 0.16 ^ab^
	150	2.34 ± 0.01 ^b^	0.44 ± 0.02 ^b^	0.14 ± 0.04 ^abc^	4.60 ± 0.01 ^c^	nd	0.82 ± 0.01 ^cd^	14.48 ± 0.18 ^b^	17.03 ± 0.04 ^c^	5.25 ± 0.35 ^ab^	2.37 ± 0.01 ^c^
BSG-F PLE	130	1.87 ± 0.06 ^d^	0.56 ± 0.03 ^a^	0.03 ± 0.00 ^d^	0.77 ± 0.11 ^g^	nd	0.63 ± 0.00 ^e^	7.42 ± 0.15 ^e^	15.79 ± 0.19 ^d^	3.96 ± 0.14 ^cd^	1.76 ± 0.33 ^d^
	150	2.60 ± 0.03 ^a^	0.44 ± 0.01 ^b^	0.08 ± 0.02 ^bcd^	5.58 ± 0.02 ^b^	nd	0.89 ± 0.03 ^c^	15.99 ± 0.07 ^a^	15.06 ± 0.22 ^e^	6.08 ± 0.63 ^a^	3.40 ± 0.07 ^b^
BSG MAE	130	0.68 ± 0.02 ^g^	0.15 ± 0.01 ^d^	0.05 ± 0.00 ^cd^	nd	nd	1.75 ± 0.05 ^a^	4.49 ± 0.02 ^f^	2.46 ± 0.03 ^h^	0.81 ± 0.14 ^ef^	2.39 ± 0.04 ^c^
	150	0.82 ± 0.03 ^e^	0.23 ± 0.02 ^c^	0.23 ± 0.04 ^a^	nd	nd	0.68 ± 0.01 ^e^	10.74 ± 0.20 ^d^	4.78 ± 0.07 ^f^	1.29 ± 0.06 ^e^	4.16 ± 0.09 ^a^
BSG-F MAE	130	2.21 ± 0.00 ^c^	0.52 ± 0.00 ^a^	0.08 ± 0.01 ^bcd^	3.09 ± 0.02 ^f^	nd	1.22 ± 0.07 ^b^	7.86 ± 0.16 ^e^	21.45 ± 0.09 ^a^	3.45 ± 0.15 ^d^	1.30 ± 0.00 ^d^
	150	2.24 ± 0.04 ^bc^	0.24 ± 0.00 ^c^	0.11 ± 0.04 ^bcd^	3.87 ± 0.03 ^d^	nd	0.62 ± 0.01 ^e^	13.20 ± 0.02 ^c^	20.77 ± 0.35 ^b^	4.67 ± 0.36 ^bc^	2.53 ± 0.02 ^c^

C: control; F: fermented; BSG: brewer’s spent grain; PLE: pressurized liquid extraction; MAE: microwave-assisted extraction; T: extraction temperature; 4-HBA: 4-hydroxybenzoic acid; *p*-CA: *p*-coumaric acid; *trans*-FA: *trans*-ferulic acid. Results are expressed as mean ± standard deviation (*n* = 2) in µg/g BSG or mg/g BSG. Different lowercase letters within the same column indicate significant differences among samples (*p* < 0.05). nd: not detected.

**Table 2:** Comparison between simple emulsion (SE) and double emulsion (DE) techniques on average particle diameter (Dp), polydispersity index (PDI), and encapsulation efficiency (EE).

Emulsion Type	Extract	Dp (nm)	PDI	EE (%)
SE	-	231 ± 11 ^b^	0.175 ± 0.02 ^ab^	-
	BSG	251 ± 31 ^ab^	0.167 ± 0.05 ^ab^	34.3 ± 5.6 ^a^
	BSG-F	260 ± 9 ^ab^	0.147 ± 0.01 ^ab^	28.4 ± 2.8 ^a^
SE+L	BSG	223 ± 8 ^b^	0.132 ± 0.003 ^b^	23.2 ± 4.8 ^a^
	BSG-F	225 ± 1 ^b^	0.149 ± 0.020 ^ab^	30.7 ± 7.6 ^a^
SE-E	BSG	254 ± 1 ^ab^	0.212 ± 0.010 ^a^	26.3 ± 3.8 ^a^
	BSG-F	216 ± 1 ^b^	0.129 ± 0.007 ^b^	25.9 ± 2.6 ^a^
DE	-	239 ± 1 ^b^	0.204 ± 0.003 ^ab^	-
	BSG	294 ± 2 ^a^	0.194 ± 0.020 ^ab^	33.9 ± 5.3 ^a^
	BSG-F	288 ± 3 ^a^	0.203 ± 0.007 ^ab^	34.7 ± 5.8 ^a^

Values refer to the average of duplicate experiments. Different letters mean a significant difference (*p* < 0.05) in the same column. SE: simple emulsion; SE+L: simple emulsion with soy lecithin; SE-E: simple emulsion with less extract; DE: double emulsion; BSG: unfermented BSG extract; BSG-F: fermented BSG extract.

## Data Availability

The original contributions presented in this study are included in the article and the Supplementary Materials. Further inquiries can be directed to the corresponding author.

## Supplementary Materials

The following supporting information can be downloaded at https://www.mdpi.com/article/10.3390/plants15192988/s1. Figure S1: DSC curve of carnauba raw wax; Figure S2: Fermentation kinetics. (**A**) Reducing compounds (Folin-Ciocalteu) and (**B**) antioxidant capacity (DPPH). Values are mean ± standard deviation (n = 3). Different letters mean a significant difference (*p* < 0.05); Figure S3: Extract yield with pressurized liquid and microwave-assisted extraction. BSG: Brewers spent grain; C: control; PLE: pressurized liquid extraction; MAE: microwave assisted extraction; F: fermented; Figure S4: Antioxidant capacity (DPPH) expressed as µmol Trolox equivalent (TE) in relation to the extract (**A**) and in relation to BSG (**B**). Results are expressed as mean ± SD (n = 3). C: control; PLE: pressurized liquid extraction; MAE: microwave-assisted extraction; F: fermented. Different lowercase letters mean a statistical significance (*p* < 0.05) between PLE extracts and capital letters between MAE extracts, and * compares C samples; Table S1: Multiple reaction monitoring (MRM) conditions used for the targeted analysis of phenolic compounds; Table S2: Effect of crodamol content and ultrasonic emulsification conditions (amplitude and time) on average diameter particle (Dp) and polydispersity index (PDI) of blank carnauba wax nanoparticles in simple emulsion; Table S3: Melting and crystallization temperatures (T) and enthalpies (ΔH) of carnauba wax (CW).
