# Supplementary material for: Integrated Bioprocessing and Nanoencapsulation of Brewer’s Spent Grain Phenolic Compounds: A Sustainable Valorization Strategy
Source: Plants (Basel). 2026 Sep 30;15(19):2988. doi: 10.3390/plants15192988 (PMC13666861; doi:10.3390/plants15192988)
Supplement: Supplementary file 1 [file plants-15-02988-s001.zip › plants-4527602-supplementary.pdf]

Supplementary material

Differential Scanning Calorimetry (DSC)

Carnauba raw wax was analyzed by Differential Scanning Calorimetry under a nitrogen atmosphere (20 mL/min) at a heating rate of 10 °C/min on a Jade DSC (Perkin Elmer) calorimeter. The phase change latent heat (ΔH_m) and the melting temperature (T_m) were recorded from the second heating ramp from 0 °C to 110 °C.

Supplementary Figures

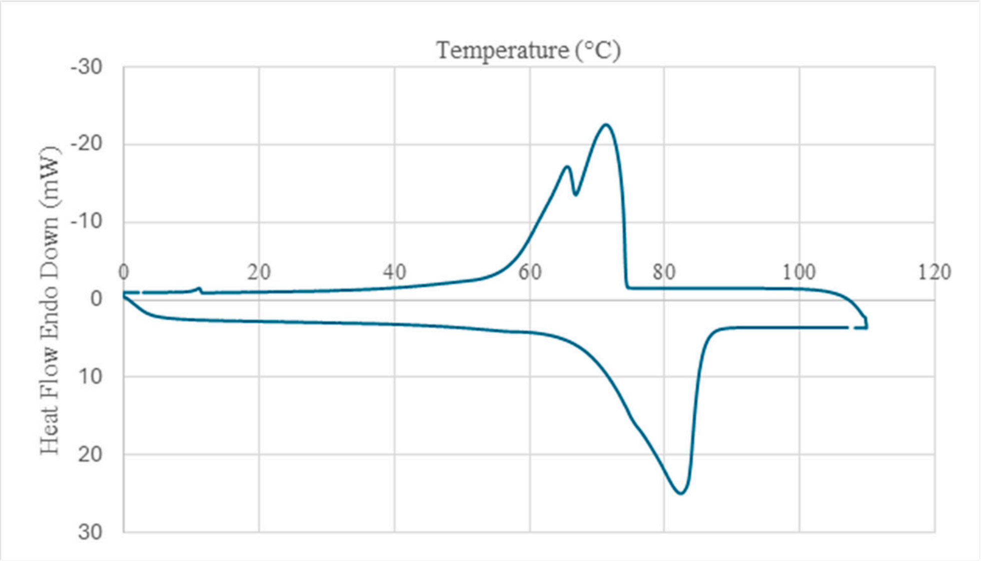

Figure S1. DSC curve of carnauba raw wax.

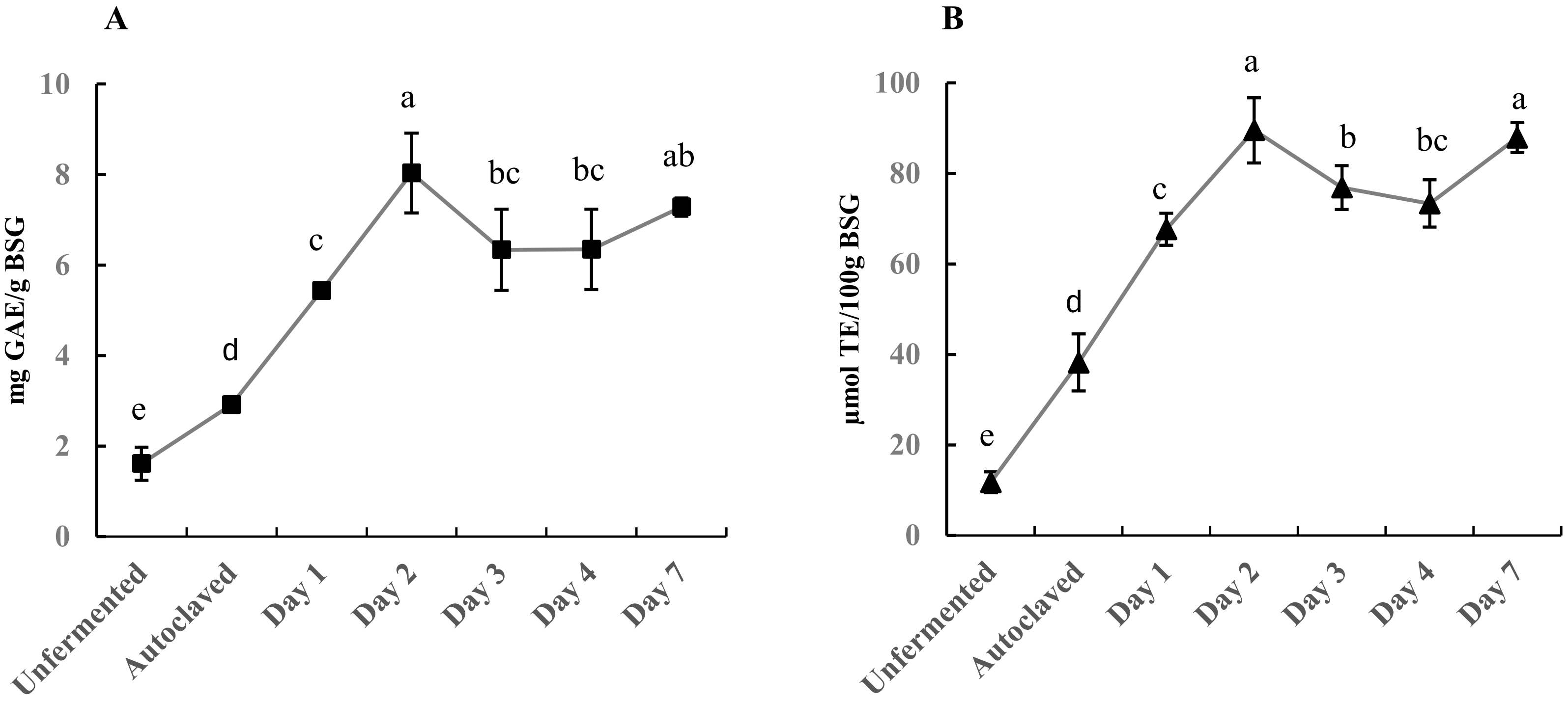

Figure S2. Fermentation kinetics. (A) Reducing compounds (Folin-Ciocalteu) and (B) antioxidant capacity (DPPH). Values are mean ± standard deviation (n=3). Different letters mean a significant difference ($p < 0.05$).

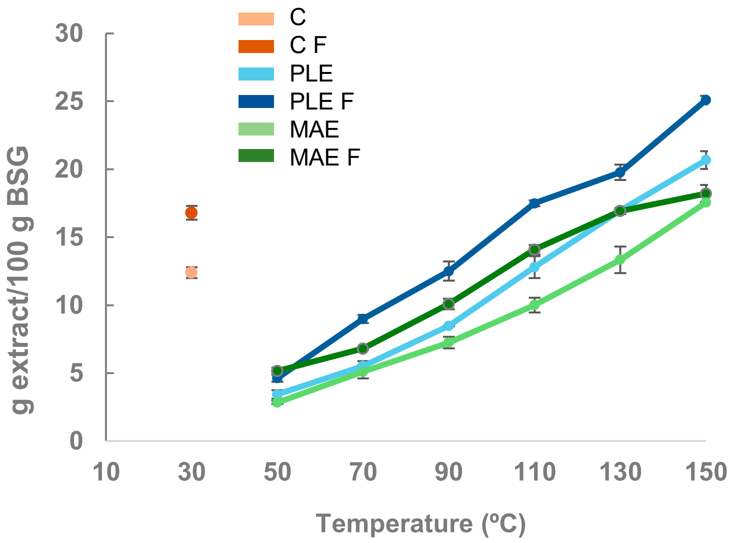

Figure S3. Extract yield with pressurized liquid and microwave-assisted extraction. BSG: Brewers spent grain; C: control; PLE: pressurized liquid extraction; MAE: microwave assisted extraction; F: fermented.

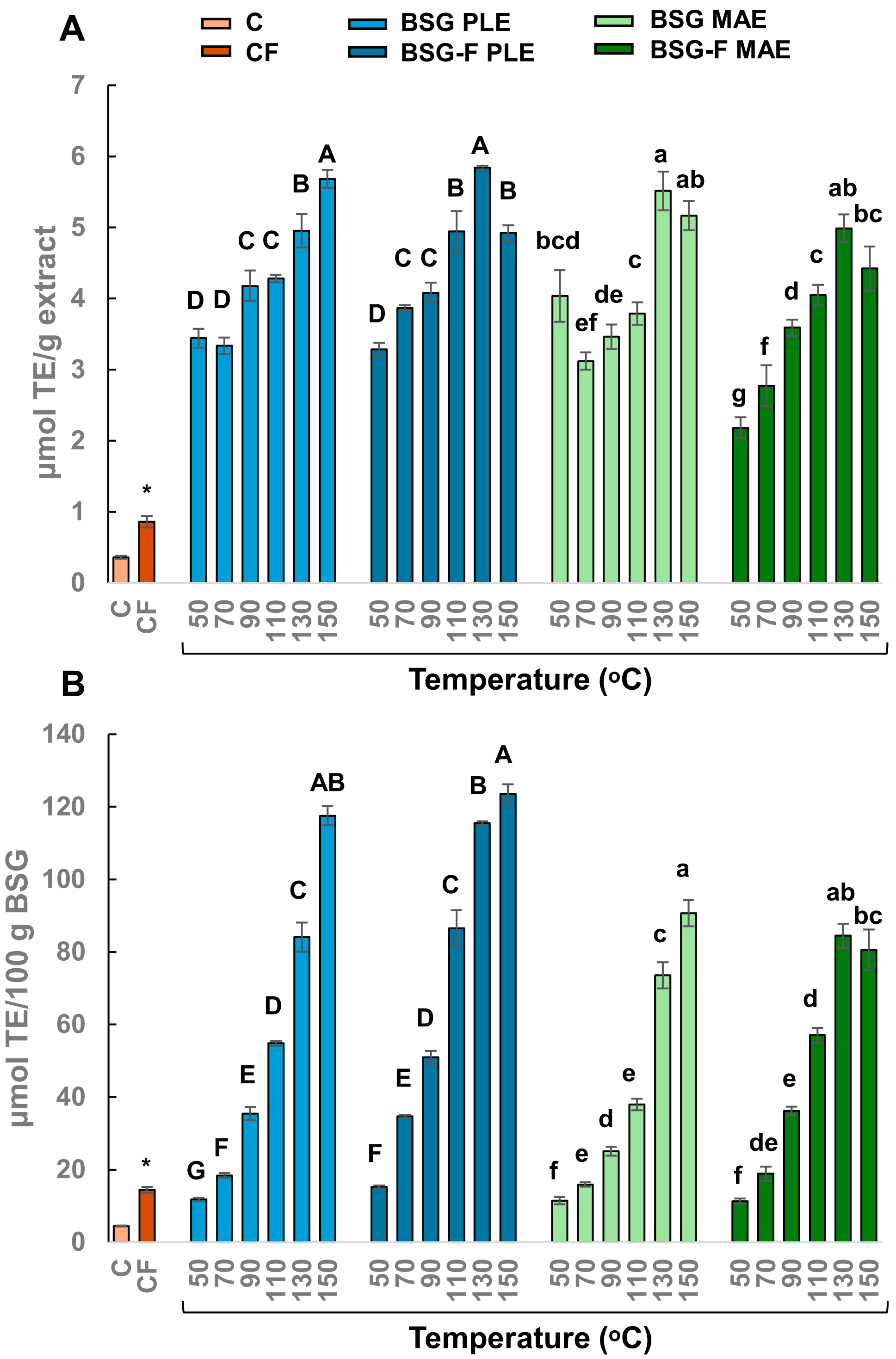

Figure S4. Antioxidant capacity (DPPH) expressed as μmol Trolox equivalent (TE) in relation to the extract (A) and in relation to BSG (B). Results are expressed as mean ± SD (n=3). C: control; PLE: pressurized liquid extraction; MAE: microwave-assisted extraction; F: fermented. Different lowercase letters mean a statistical significance ($p < 0.05$) between PLE extracts and capital letters between MAE extracts, and * compares C samples.

Supplementary Tables

Table S1. Multiple reaction monitoring (MRM) conditions used for the targeted analysis of phenolic compounds.

**Compound name**	**Molecular formula**	**Adduct type**	**RT (min)**	**Precursor ion (*m/z*)**	**Cone voltage (V)**	**Transition ion 1 (*m/z*)**	**Collision energy 1 (eV)**	**Transition ion 2 (*m/z*)**	**Collision energy 2 (eV)**
4-Hydroxybenzoic acid	$C_7H_6O_3$	$[M-H]^-$	5.07	137	25	93*	14	65	22
Caffeic acid	$C_9H_8O_4$	$[M-H]^-$	5.94	179	35	117	20	135*	15
Catechin	$C_{15}H_{14}O_6$	$[M-H]^-$	5.19	289	8	123*	38	202	22
Cyanidin chloride	$C_{15}H_{11}O_6$	$[M]^+$	12.71	287	35	213	31	137*	35
Ferulaldehyde	$C_{10}H_{10}O_3$	$[M+H]^+$	5.19	179	25	147	22	161*	15
Gallic acid	$C_7H_6O_5$	$[M-H]^-$	2.24	169	50	79*	24	97	16
p-Coumaric acid	$C_9H_8O_3$	$[M-H]^-$	7.69	163	48	93	30	119*	26
trans-Ferulic acid	$C_{10}H_{10}O_4$	$[M-H]^-$	8.45	193	60	134*	16	178	12
Tyrosol	$C_8H_{10}O_2$	$[M+H]^+$	3.27	139	25	139	0	121*	15
Vanillic acid	$C_8H_8O_4$	$[M-H]^-$	5.86	167	25	108	25	152*	10
Vanillin	$C_8H_8O_3$	$[M-H]^-$	7.46	151	20	92	20	136*	10

Abbreviations: RT, retention time; *m/z*, mass-to-charge ratio. The adducts "$[M-H]^+$" and "$[M-H]^-$" indicate positive and negative ionization modes, respectively. MRM transitions marked with an asterisk (*) correspond to the quantifier ions used for compound quantification.

Table S2. Effect of crodamol content and ultrasonic emulsification conditions (amplitude and time) on average diameter particle (Dp) and polydispersity index (PDI) of blank carnauba wax nanoparticles in simple emulsion.

Formulation	CW (g)	Crodamol (g)	Ultrasonic Emulsification		Water 4 °C	Dp (nm)	PDI
			Amplitude (%)	Time (s)			
Mini-1	1.0	-	50	60	-	115 ± 1	0.530 ± 0.008
Mini-2	0.9	0.1	50	60	90 ml	154 ± 6	0.320 ± 0.006
Mini-3	0.9	0.1	50	120	90 ml	270 ± 1	0.132 ± 0.001
Mini-4	0.9	0.1	50	150	90 ml	246 ± 2	0.170 ± 0.001
Mini-5	0.9	0.1	50	210	90 ml	264 ± 1	0.200 ± 0.002
Mini-6	0.9	0.1	70	120	90 ml	223 ± 3	0.074 ± 0.007

CW: Carnauba wax; Mini: miniemulsion formulation tested.

Table S3. Melting and crystallization temperatures (T) and enthalpies (ΔH) of carnauba wax (CW).

	**Melting**		**Crystallization**		
	$\boldsymbol{T_m}$ (°C)	$\boldsymbol{\Delta H_m}$ (J/g)	$\boldsymbol{T_\alpha}$ (°C)	$\boldsymbol{T_\beta}$ (°C)	$\boldsymbol{\Delta H_c}$ (J/g)
**CW**	82.40	171.61	65.64	71.42	-173.86
